# Prognostic impact of immunohistopathologic features in definitive radiation therapy for nasopharyngeal cancer patients

**DOI:** 10.1093/jrr/rrz071

**Published:** 2019-12-10

**Authors:** Naoya Murakami, Taisuke Mori, Yuko Kubo, Seiichi Yoshimoto, Kimiteru Ito, Yoshitaka Honma, Takao Ueno, Kenya Kobayashi, Hiroyuki Okamoto, Narikazu Boku, Kana Takahashi, Koji Inaba, Kae Okuma, Hiroshi Igaki, Yuko Nakayama, Jun Itami

**Affiliations:** 1 Department of Radiation Oncology, National Cancer Center Hospital, Japan; 2 Department of Pathology and Clinical Laboratories, National Cancer Center Hospital, Japan; 3 Department of Diagnostic Radiology, National Cancer Center Hospital, 5-1-1 Tsukiji, Chuo-ku, Tokyo, Japan; 4 Department of Head and Neck Surgery, National Cancer Center Hospital, Japan; 5 Department of Head and Neck Medical Oncology, Head and Neck Medical Oncology Division, National Cancer Center Hospital, Japan; 6 Department of Oral Health and Diagnostic Sciences, National Cancer Center Hospital, Japan

**Keywords:** nasopharyngeal cancer, intensity modulated radiation therapy, chemoradiation therapy, immunohistopathologic features, EpCAM

## Abstract

Our previous study by Murakami N, Mori T, Nakamura S, Yoshimoto S, Honma Y, Ueno T, Kobayashi K, Kashihara T, Takahashi K, Inaba K, Okuma K, Igaki H, Nakayama Y, Itami J. (J Radiat Res. 2019 Jul 30. pii: rrz053. doi: 10.1093/jrr/rrz053. [Epub ahead of print]) showed that strong expression of epithelial cell adhesion molecule (EpCAM) was associated with radiation resistance in head and neck squamous cell cancer patients (SCC). In this study, the prognostic impact of histopathologic features including EpCAM for nasopharyngeal cancer (NPC) patients was investigated. Since 2009, our institution has performed chemoradiation for locally advanced NPC patients with intensity modulated radiation therapy (IMRT). Tri-weekly adjuvant cisplatin (CDDP, 80 mg/m^2^) was administered concurrently with definitive radiation therapy 70 Gy in 35 fractions. One month after radiation therapy, adjuvant chemotherapy of three cycles of CDDP/5 fluorouracil (5-FU) was administered. Using a pretreatment biopsy specimen, EBV-encoded small RNA *in situ* hybridization (EBER-ISH), EpCAM, p16 and p53 were assessed by immunohistochemical analysis. Between May 2009 and September 2017, 51 NPC patients received definitive radiation therapy. Five, 13, 17 and 16 patients were staged as I, II, III and IV, respectively. The median follow-up period for alive patients was 31.1 months (12.4–109.7 months). Three-year overall survival (OS), progression-free survival (PFS) and locoregional control (LRC) were 87.1, 57.1 and 85.7%, respectively. EpCAM, p16 and p53 were not associated with PFS, OS nor LRC. Three-year PFS for patients with keratinizing and non-keratinizing SCC were 25 and 60.5%, respectively (*P* = 0.033, hazard ratio 4.851 (95% confidence interval 1.321–17.814)).Prognosis of NPC patients with keratinizing SCC was worse than non-keratinizing SCC patients, suggesting a biological difference between the two types of tumor.

## INTRODUCTION

While nasopharyngeal cancer (NPC) is commonly seen in China [1, 2] and Southeast Asia [3, 4], it is rarely seen in the rest of the non-endemic world including Japan [5].

The development of non-keratinizing squamous cell carcinoma (SCC), the predominant type of NPC, is related to Epstein-Barr virus (EBV) infection, and EBV screening is used for disease detection in endemic regions [6, 7].

Intensity modulated radiation therapy (IMRT) has been used for NPC patients to protect major salivary glands, spinal cord, constrictor muscle and thyroid gland to maintain the patient’s later organ function and contribute to maintaining quality of life [8–10].

The epithelial cell adhesion molecule (EpCAM) or CD326 is a 30–40 kDa glycosylated type I membrane protein [11]). EpCAM expression is restricted to epithelial tissues in adults [12] and functions as an intercellular cell adhesion molecule. It is related to cellular signaling, cell migration, differentiation and proliferation [13]. In pathologic circumstances such as malignancies and inflammatory responses, its expression can be increased [14]. Our previous report demonstrated that overexpression of EpCAM was associated with local recurrence after definitive radiation therapy for early-stage glottic cancer patients [15], and in other head and neck sites that consisted mostly of hypopharynx, oropharynx and larynx, we showed that EpCAM was a prognostic factor [16]. Other research also showed that EpCAM was a prognostic biomarker in head and neck cancer [17, 18]. However, the prognostic role of EpCAM is undetermined in NPC [19]. In this single institutional retrospective study, authors investigated the relationship between clinicopathologic features including EpCAM and clinical outcome for NPC patients treated with definitive chemoradiation therapy.

## MATERIALS AND METHODS

NPC patients who were treated by definitive radiation therapy were included in this study. Staging workup included a complete history and physical examination, a complete blood count, serum biochemistry tests, computed tomography (CT) of the neck, thorax and upper abdomen, and magnetic resonance imaging (MRI) of the neck. Tumors were staged by the 7th edition of the tumor node metastasis (TNM) classification system. Patients with distant metastasis or who only received a palliative radiation dose were excluded from the study.

### Chemotherapy

Since 2009, concurrent chemoradiation therapy (cCRT), which is standard care for locally advanced NPC, has been performed in our hospital. This is carried out according to the Japan Clinical Oncology Study 1015 [UMIN000005448UMIN000005448], a phase II study of IMRT with concurrent chemotherapy for locally advanced NPC, concurrent chemoradiotherapy with two-step IMRT and three cycles of cisplatin (80 mg/m^2^, day 1, every 3 weeks) followed by three cycles of adjuvant cisplatin (CDDP) (70 mg/m^2^, day 1) and 5-fluorouracil (5-FU) (700 mg/m^2^, days 1–5) every 4 weeks. Patients with Stage I disease, over 80 years old or with renal dysfunction received radiation therapy alone. Since July 2017, because a phase III clinical trial demonstrated better 3-year progression-free survival (PFS) with induction chemotherapy followed by cCRT compared with cCRT followed by adjuvant CDDP and 5-FU [20–22], T4 or N3 patients were offered induction docetaxel (70 mg/m^2^ on day 1), CDDP (70 mg/m^2^ on day 1) and 5-FU (750 mg/m^2^ from day 1 to day 5) (DCF) followed by cCRT.

### Radiation therapy

Before starting radiation therapy, every patient went through a dental check-up and a mouthpiece was created. Fixation masks were created with the mouthpiece in place. Contrast-enhanced CT and MRI scans were taken for planning. Radiation therapy was delivered in a conventional 2 Gy/fraction schedule. The total dose was 70 Gy/35 fractions in 7 weeks and a secondary boost plan started after 46 Gy. Another CT scan was taken for planning a secondary boost plan adapting to tumor shrinkage and body habitus change. CTV_46Gy_ included primary lesion, nasopharyngeal area, enlarged lymph nodes, bilateral level II, III, IV, V, supraclavicular and retropharyngeal lymph node area [23]. A secondary planning CT scan was taken and an adaptive plan was created with CTV_70Gy_ including enlarged lymph node, primary lesion and nasopharyngeal area.

Supportive care measures, such as gargling with an anti-inflammatory drug, acetaminophen, opioids and liquid nutrition, were prescribed to cope with severe radiation mucositis. Oral nutritional supplements were prescribed to help maintain the patient’s body weight. When weight reduction of >10% from baseline was found, gastrostomy was encouraged.

### Histopathologic analysis

Sections (4-μm thick) from a representative block of each pretreatment biopsy specimen were routinely deparaffinized. The sections were subjected to hematoxylin-eosin and immunohistochemical staining. Immunohistochemical staining was performed with the following primary antibodies: EpCAM (1:200, ab7504, BerEP4; Abcam, Cambridge, MA); p53 (1:400, DO-7, Dako, Caprinteria, CA); and p16 (1:50, p16ink4a, G175–405; BD Biosciences, San Jose, CA). Each section was exposed to 0.3% hydrogen peroxide for 15 min to block endogenous peroxidase activity. For staining, an automated stainer (Dako, Caprinteria, CA) was used according to the protocol of the manufacturer. The ChemMate EnVision method (Dako, Caprinteria, CA) was used for detection. Appropriate positive and negative controls were used for each antibody. BerEP4 positivity, one of the antibodies against EpCAM, was defined as follows: minus (−), no expression; one plus (+), weak to moderate expression; and two plus (++), intense expression. Strong expression of nuclear p53 (accumulation) or no expression (missense of exons 5–9 of p53, where most known abnormalities occur) was considered to indicate a p53 gene mutation [[19, 20]]; in the rest of cases, the specimen was considered not to have a p53 mutation by immunohistochemistory. Only tumors with p16 expression both in the nucleus and cytoplasm were classified as p16 positive. For EBV detection, we used the paraffin section RNA *in situ* hybridization (ISH) technique with digoxigenin-labeled riboprobes for demonstrating EBV-encoded small RNAs (EBERs), which are EBV gene products that are transcribed during latent EBV infection.

### Statistics

Overall survival (OS) was calculated from the start of radiation therapy until the date of death from any cause or censored at the last follow-up visit. PFS was calculated from the start of radiation therapy to the date of any disease relapse or censored at the last follow-up visit or the date of death from any cause. Locoregional control (LRC) was calculated from the start of radiation therapy until the date of locoregional relapse or censored at the last follow-up visit or the date of death from any cause. The survival curves were estimated by using the Kaplan-Meier method and the differences were assessed by the log-rank test. A *P* value < 0.05 was considered statistically significant. Factors with *P* value < 0.05 were further analyzed by multivariate analysis using Cox regression analysis. Cox proportional-hazards models were used to estimate hazard ratios. All analyses were performed using IBM SPSS Statistics (version 18.0; SPSS, Inc., Chicago,IL). All procedures performed in the study involving human participants were approved and in accordance with the ethical standards of the institutional research committee (approval number is 2017–091) and the 1964 Helsinki declaration and its later amendments or comparable ethical standards.

## RESULTS

From May 2009 to September 2017, 59 consecutive NPC patients were referred to our department. Eight patients were excluded because of the following reasons and the remaining 51 were included in the analysis: 6 had distant metastasis, 1 had a prior history of radiation therapy in the head and neck region and could not receive standard radiation therapy, 1 committed suicide soon after the end of the irradiation. Patients demographics are summarized in Table 1. Five, 13, 17 and 16 patients were classified into Stage I, II, III and IVA, respectively. 92.1% of patients were non-keratinizing type SCC and 85.1% of the non-keratinizing type were positive for EBER-ISH. There was a discrepancy between EBER-ISH and pathologic findings; there were 8 patients with EBER-ISH negative tumor. However, 5 out of these 8 patients were non-keratinizing SCC. It was found that EBER-ISH negative patients were not always keratinizing SCC. More than half of patients (58.8%) had overexpression of BerEP4. Five patients were positive for p16 and 3 of them were also positive for EBER-ISH. Table 2 summarizes the treatment details. Two patients received induction DCF followed by cCRT. 82.3% of patients received cCRT followed by adjuvant FP. The median cycles of CDDP administered concurrently with RT was 3 cycles. The median cycles of adjuvant FP administered after RT was 2 cycles.

**Table 1:** Patient characteristics

Sex		
	Male	36
	Female	15
Median age, years (range)		56 (12–83)
Histology		
	Non-keratinizing	47
	Keratinizing	4
T stage		
	1	21
	2	11
	3	11
	4	8
N stage		
	0	9
	1	13
	2	18
	3	11
Stage		
	I	5
	II	13
	III	17
	IV	16
p16		
	Negative	42
	Positive	5
	Unknown	4
p53		
	Wild type	28
	Mutated	19
	Unknown	4
BerEP4		
	Overexpression	30
	Non-overexpression	17
	Unknown	4
EBER-ISH		
	EBV uninfected pattern	8
	EBV infected pattern	40
	Unknown	3

EBV = Epstein-Barr virus,

EBER-ISH = EBV-encoded small RNA *in situ* hybridization.

**Table 2:** Treatment details

Induction chemotherapy		
	Yes	2
	No	49
Concurrent chemotherapy		
	Yes	42
	No	9
	Median administered cycles	3 (0–3)
Adjuvant chemotherapy		
	Yes	28
	No	23
	Median administered cycles	2 (0–3)
IMRT method		
	Conventional IMRT	15
	VMAT	36
Median total radiation dose (Gy)		70 (59.4–82)
	
	

IMRT = intensity modulated radiation therapy,

VMAT = volumetric modulated arc therapy.

**Fig. 1:**
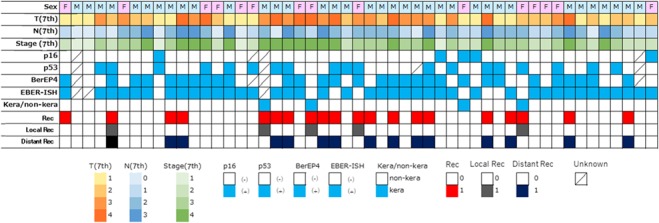
Clinicopathologic status and clinical outcome: the landscape of clinical and immunohistochemical status across the 51 nasopharyngeal squamous carcinomas. Rec = recurrence, F = female, M = male.

Figure 1 provides an overview of clinicopathologic features and clinical outcome. Median follow-up period for alive patients was 31.1 months (12.4–109.7 months). Three-year OS, PFS and LRC were 87.1, 57.1 and 85.7%, respectively. There were six in-field recurrences; 5 patients experienced a local recurrence in their primary site and 3 of them were keratinizing SCC. One patient received salvage surgery which resulted in the second relapse in the local site. Another patient received repeated irradiation with stereotactic radiotherapy using small fields and the disease is fortunately under control without any severe sequelae. The last patient received salvage neck dissection for residual neck lymph node and viable cells were confirmed by final pathology and since then this patient remains disease-free.

The results of uni- and multi-variate analysis are summarized in Table 3. BerEP4, p16 and p53 were not associated with PFS, OS nor LRC. T stage, N stage, Stage, and pathologic features were associated with PFS, and pathologic features and EBER-ISH were associated with OS and LRC, respectively. Patients <56 years old showed a trend toward better OS (*P* = 0.071). Multiple variate analysis found that T stage and histopathological features were independent factors associated with PFS, and pathologic features were also found to be a significant factor for OS and LRC.

**Table 3:** Clinicopathologic factors related to progression-free survival (PFS), overall survival (OS) and locoregional control (LRC)

				*P* in uni.	*P* in multi.	Hazard ratio (95% CI)
PFS		3-year PFS				
Age, years	<56 vs ≥56	62.8	51.6	0.133		
T stage	T1–3 vs T4	67.0	12.5	<0.001^*^	<0.001^*^	5.681 (2.150–14.925)
N stage	N0–1 vs N2–3	72.9	45.6	0.050^*^		
Stage	I-II vs III-IV	83.3	44.1	0.028^*^		
Concurrently given CDDP (Stages II-IV)	3 cycles vs <3 cycles	52.3	50.1	0.959		
Pathological features	Keratinizing vs non-keratinizing	25.0	60.5	0.033^*^	0.017^*^	4.851 (1.321–17.814)
p53	Mutated vs un-mutated	53.9	61.6	0.816		
EBER-ISH	EBV uninfected vs infected type	33.3	61.2	0.252		
p16	Positive vs negative	100.0	51.4	0.333		
BerEP4	Intensive expression vs others	51.2	63.7	0.477		
OS						
Age	<56 vs ≥56	95.7	79.7	0.071		
T stage	T1–3 vs T4	89.6	72.9	0.157		
N stage	N0–1 vs N2–3	100.0	77.5	0.083		
Stage	I-II vs III-IV	100.0	80.2	0.162		
Concurrently given CDDP (Stages II-IV)	3 cycles vs <3 cycles	87.8	84.1	0.595		
Pathological features	Keratinizing vs non-keratinizing	50.0	90.4	0.002^*^	0.012^*^	9.077 (1.633–50.450)
p53	Mutated vs un-mutated	78.5	96.3	0.119		
EBER-ISH	EBV uninfected vs infected type	62.5	93.8	0.034^*^		
p16	Positive vs negative	100.0	86.8	0.516		
BerEP4	Intensive expression vs others	84.8	94.1	0.490		
LRC						
Age, years	<56 vs ≥56	100.0	72.6	0.013^*^		
T stage	T1–3 vs T4	85.6	87.5	0.877		
N stage	N0–1 vs N2–3	90.9	81.3	0.570		
Stage	I-II vs III-IV	88.9	83.6	0.870		
Concurrently given CDDP (Stage II-IV)	3 cycles vs <3 cycles	90.1	80.4	0.262		
Pathological features	Keratinizing vs non-keratinizing	25.0	92.0	<0.001^*^	0.001^*^	16.045 (3.181–80.931)
p53	Mutated vs un-mutated	78.2	92.6	0.464		
EBER-ISH	EBV uninfected vs infected type	46.7	94.9	0.02^*^		
p16	Positive vs negative	100.0	84.8	0.272		
BerEP4	Intensive expression vs others	86.9	86.3	0.682		

CDDP = cisplatin,

EBV = Epstein-Barr virus,

EBER-ISH = EBV-encoded small RNA *in situ* hybridization,

uni. = univariate analysis,

multi. = multivariate analysis,

p values less than 0.05 were considered to indicate statistical significance and were marked with asterisk.

Similar analysis was performed for patients only with non-keratinizing SCC (Table 4). Although it did not reach statistical significance, overexpression of BerEP4 was associated with worse PFS (*P* = 0.089). It was also found that mutated p53 was associated with worse OS (*P* = 0.018).

**Table 4:** Clinicopathologic factors related to progression-free survival (PFS), overall survival (OS) and locoregional control (LRC) for non-keratinizing squamous cell carcinoma

				*P* in uni.	*P* in multi.	Hazard ratio (95% CI)
PFS		3-year PFS				
Age, years	<56 vs ≥56	62.8	56.9	0.267		
T stage	T1–3 vs T4	70.3	14.3	0.001^*^	0.002^*^	4.975 (1.792–13.889)
N stage	N0–1 vs N2–3	72.0	51.6	0.145		
Stage	I-II vs III-IV	82.4	49.1	0.074		
Concurrently given CDDP (Stage 2–4)	3 cycles vs < 3 cycles	52.3	72.9	0.120		
p53	Mutated vs un-mutated	57.7	62.8	0.963		
EBER-ISH	EBV uninfected vs infected type	30.0	61.2	0.208		
p16	Positive vs negative	100.0	54.6	0.341		
BerEP4	Intensive expression vs others	49.7	74.2	0.089		
OS						
Age, years	<56 vs ≥56	95.7	85.0	0.171		
T stage	T1–3 vs T4	91.4	83.3	0.522		
N stage	N0–1 vs N2–3	100.0	83.0	0.215		
Stage	I-II vs III-IV	100.0	85.1	0.340		
Concurrently given CDDP (Stages II-IV)	3 cycles vs < 3 cycles	84.1	100.0	0.347		
p53	Mutated vs un-mutated	77.7	100.0	0.018^*^	0.25	0.008 (0–28.888)
EBER-ISH	EBV uninfected vs infected type	60.0	93.8	0.027^*^		
p16	Positive vs negative	100.0	88.7	0.828		
BerEP4	Intensive expression vs others	84.2	100.0	0.138		
LRC						
Age, years	<56 vs ≥56	100.0	82.8	0.062	0.362	8.771 (0.082–1000)
T stage	T1–3 vs T4	90.6	100.0	0.473		
N stage	N0–1 vs N2–3	90.5	92.9	0.460		
Stage	I-II vs III-IV	88.2	93.8	0.287		
Concurrently given CDDP (Stages II-IV)	3 cycles vs < 3 cycles	90.1	94.1	0.820		
p53	Mutated vs un-mutated	85.0	96.0	0.621		
EBER-ISH	EBV uninfected vs infected type	50.0	94.9	0.377		
p16	Positive vs negative	100.0	90.3	0.896		
BerEP4	Intensive expression vs others	86.2	100.0	0.339		

EBV = Epstein-Barr virus,

EBER-ISH = EBV-encoded small RNA *in situ* hybridization,

uni. = univariate analysis,

multi. = multivariate analysis.

Table 5 summarizes the characteristics of patients who experienced disease progression. Along with the results in Table 3, patients with disease progression had a more advanced Stage. Similar to the findings of Table 4, the majority of patients with disease progression had overexpression of BerEP4.

**Table 5:** Characteristics of patients who experienced disease progression (*n* = 20)

Sex		
	Male	16
	Female	4
Median age, years (range)		63 (18–79)
Histology		
	Non-keratinizing	17
	Keratinizing	3
T stage		
	1	4
	2	2
	3	7
	4	7
N stage		
	0	2
	1	3
	2	9
	3	6
Stage		
	I	0
	II	2
	III	7
	IV	10
p16		
	Negative	19
	Positive	0
	Unknown	1
p53		
	Wild type	10
	Mutated	8
	Unknown	2
BerEP4		
	Overexpression	14
	Non-overexpression	5
	Unknown	1
EBER-ISH		
	EBV uninfected pattern	5
	EBV infected pattern	14
	Unknown	1
Treatment		
	RT alone	3
	Induction CT followed by cCRT	1
	cCRT	16

EBV = Epstein-Barr virus,

EBER-ISH = EBV-encoded small RNA *in situ* hybridization,

RT = radiation therapy,

CT= chemotherapy,

cCRT = concurrent chemoradiotherapy.

### Toxicities

Acute and late toxicities are summarized in Table 6. Of patients, 13.7 and 9.8% had grade 3 or higher dermatitis and mucositis, respectively; 29.4% had gastrostomy or total parental nutrition (TPN); and 7.8 and 1.9% experienced late radiation-related hypothyroidism and osteoradionecrosis, respectively. All patients eventually became independent of gastrostomy and TPN several months after completion of the treatment.

**Table 6:** Treatment-related toxicities

Acute toxicities		
	Dermatitis ≥3	7 (13.7%)
	Mucositis ≥3	5 (9.8%)
	Gastrostomy or TPN utilization	15 (29.4%)
		
Late toxicities		
	Secondary hypothyroidism	4 (7.8%)
	Osteoradionecrosis	1 (1.9%)

TPN = total parenteral nutrition.

## DISCUSSION

This study is in a relatively large cohort of NPC patients treated by cCRT using IMRT in a non-NPC endemic country. In our previous report, EpCAM was associated with radiation response for early-stage glottic cancer patients [15] as well as other head and neck sites that consisted mostly of hypopharynx, oropharynx and larynx [16]. When focused only on NPC cancer patients, it was found that EpCAM was not associated with prognosis after primary radiation therapy. This result suggested that although NPC is histologically classified as the same as SCC, the development of SCC in NPC might be different from SCC from other sites of the head and neck region. However, when we focused only on non-keratinizing SCC, overexpression of EpCAM indicated worse PFS, which was in line with other site head and neck SCC. Therefore, it was suggested that overexpression of EpCAM might be a negative prognostic factor for NPC with non-keratinizing SCC. On the other hand, EBV or keratinizing pathologic features were found to be associated with OS with statistical significance, which was in line with previous findings [24, 25]. These results suggest that carcinogenesis of EBV non-related NPC might be, again, different from that of EBV-related NPC and require a specific treatment strategy because the prognosis of EBV non-related NPC with current cCRT is quite poor. Investigating actionable mutation for EBV non-related NPC and combining targeted agents and IMRT would be an attractive alternative strategy for this group of patients with poor prognosis.

Although T or N stage was associated with PFS, it was found that LRC was favorable regardless of T or N stage, suggesting that if CTV was determined correctly and enough dose was delivered by IMRT, favorable LRC could be expected with definitive (chemo) radiation therapy. The strongest adverse factor for LRC was found to be the pathological features; hazard ratio of keratinizing SCC was as much as 16.045 (95% confidence interval 3.191–80.931). However, delivering a higher dose than the current standard of 70 Gy would eventually cause subsequent devastating ulceration of the posterior wall of the nasopharynx or osteoradionecrosis of the skull base, therefore, another effective strategy should be established for controlling keratinizing NPC.

In The National Comprehensive Cancer Network (NCCN) guideline version 2.2018, concurrent CRT followed by adjuvant chemotherapy and induction chemotherapy followed by CRT are equally presented as standard therapeutic options for T1, N1–3; T2–4 any N NPC patients [26]. On the other hand, better 3-year failure-free survival was shown with induction chemotherapy followed by CRT than with concurrent CRT alone in stage III–IVB (except T3–4 N0) NPC patients in a phase 3 clinical trial [20–22]. Three-year PFS for patients with Stage III/IV was 44.1% in the current study which was lower than Chinese clinical trial results which applied induction chemotherapy followed by CRT. Since 2017, we have started to offer induction DCF followed by cCRT for T4 or N3 patients, and we expect better clinical results with the new more intensive regimen for locally advanced NPC patients in the future.

There are several limitations in our study. Because our country is not a NPC endemic country, the number of patients included in this study is small. In particular, the number of patients with keratinizing SCC was very small. Because some patients had their biopsy performed by other institutions, not all biopsy samples underwent histopathologic analysis. For HPV infection analysis, DNA itself was not analyzed in this study, on the other hand, p16 was utilized as a surrogate marker for human papilloma virus (HPV) infection. For p53 analysis, again, although it is recommended that molecular analysis should be performed, because immune-histochemical analysis cannot distinguish the several different types of mutations [27, 28], the gene itself was not analyzed in this study. Therefore, the results of this study should be interpreted with caution. The dosage of CDDP used in our institution was 80 mg/m^2^ which is lower than the standard 100 mg/m^2^. This was because patients who underwent concurrent chemoradiotherapy were managed by the Department of Radiation Oncology, and we frequently deal with elderly patients with multiple co-morbidities, therefore, the traditional intensity of chemotherapy was lowered in order to avoid chemotherapy-related acute side effects. Consequently, results derived from the lower intensity chemotherapy in this study should be interpreted with caution. Despite these limitations, important clinicopathologic information for NPC was obtained from the current study.

## CONCLUSIONS

Prognosis of patients with keratinizing SCC was worse than for non-keratinizing SCC patients, suggesting a biological difference between the two types of the tumor.

It was suggested that overexpression of EpCAM might be a negative prognostic factor for NPC with non-keratinizing SCC.
